# Transcriptome sequencing and metabolite analysis reveals the role of delphinidin metabolism in flower colour in grape hyacinth

**DOI:** 10.1093/jxb/eru168

**Published:** 2014-04-30

**Authors:** Qian Lou, Yali Liu, Yinyan Qi, Shuzhen Jiao, Feifei Tian, Ling Jiang, Yuejin Wang

**Affiliations:** ^1^College of Horticulture, Northwest A & F University, Yangling 712100, Shaanxi, PR China; ^2^Key Laboratory of Biology and Genetic Improvement of Horticultural Crops (Northwest Region), Ministry of Agriculture, Yangling, Shaanxi 712100, PR China; ^3^State Key Laboratory of Crop Stress Biology in Arid Areas, Northwest A&F University, Yangling 712100, Shaanxi, PR China; ^4^College of Forestry, Northwest A & F University, Yangling 712100, Shaanxi, PR China

**Keywords:** Colour pigmentation, cyanidin, delphinidin, flower development, *Muscari armeniacum*, transcriptome analysis.

## Abstract

Grape hyacinth (*Muscari*) is an important ornamental bulbous plant with an extraordinary blue colour. *Muscari armeniacum*, whose flowers can be naturally white, provides an opportunity to unravel the complex metabolic networks underlying certain biochemical traits, especially colour. A blue flower cDNA library of *M. armeniacum* and a white flower library of *M. armeniacum* f. album were used for transcriptome sequencing. A total of 89 926 uni-transcripts were isolated, 143 of which could be identified as putative homologues of colour-related genes in other species. Based on a comprehensive analysis relating colour compounds to gene expression profiles, the mechanism of colour biosynthesis was studied in *M. armeniacum*. Furthermore, a new hypothesis explaining the lack of colour phenotype of the grape hyacinth flower is proposed. Alteration of the substrate competition between flavonol synthase (FLS) and dihydroflavonol 4-reductase (DFR) may lead to elimination of blue pigmentation while the multishunt from the limited flux in the cyanidin (Cy) synthesis pathway seems to be the most likely reason for the colour change in the white flowers of *M. armeniacum.* Moreover, mass sequence data obtained by the deep sequencing of *M. armeniacum* and its white variant provided a platform for future function and molecular biological research on *M. armeniacum*.

## Summary

Through a combination of metabolite analysis with transcriptome sequencing, a new hypothesis was proposed to explain the lack of colour phenotype of the white variant of the blue grape hyacinth.

## Introduction

Grape hyacinth (*Muscari*) is an important ornamental bulbous plant with a unique flower shape, extraordinary blue colour, and sweet fragrance (Qi *et al.*, 2013). These quality traits are largely determined by the metabolic composition of the flower. For example, anthocyanins are the principal flower pigments in *Muscari* flowers (Mori *et al.*, 2002). It is reported that the varying shades found in the blue flowers are attributable to delphinidin (Del), while the reddish hues are attributable to cyanidin (Cy) (Qi *et al.*, 2013). Anthocyanins are among the most studied and best understood compounds in plant science, and their metabolic pathway has been extensively described (Grotewold, 2006; Tanaka *et al.*, 2008). Nevertheless, the mechanisms that control anthocyanin catabolism in different plant species are far from conclusive. It is reasonable to expect that such loss-of-colour adaptations are relatively unconstrained because they can be achieved in many ways (Clark and Verwoerd, 2011). The numerous diverse metabolic pathways by which plant compounds can be produced makes it more difficult to clarify this matter.

The increased ease and efficiency of RNA sequencing (RNA-Seq) tools will facilitate the study of the mechanisms underlying metabolite variation. However, it is still hard to imagine a direct correlation between the transcript abundance and the level of respective metabolite. After all, there are always too many variable factors to reach a clear conclusion. On the basis of metabolite analysis, a stringent logical filter for high-throughput approaches could be set up and used to identify the relevant factors and to circumvent the ambiguities resulting from the transcriptome comparison between different varieties. By choosing an integrative approach, where not only are transcript levels investigated, but also the metabolic products are compared, it is possible to gain an insight into metabolic flows, which would not be possible from transcript analysis alone. Thus the natural variation in blue *M. armeniacum* flower (the white form of *M. armeniacum*) provides opportunities for insight into complex metabolic networks and certain biochemical traits, especially colour.

In the present study, the first RNA-Seq project for *M. armeniacum* and its white variant was performed using the Illumina sequencing technique. Through a combination of chemical analysis with bioinformatics, the major metabolic pathways related to *Muscari* flower pigmentation were deduced and the candidate genes targeting the loss of pigmentation in the plants were examined.

## Materials and methods

### Plant material

**Fig. 1.**
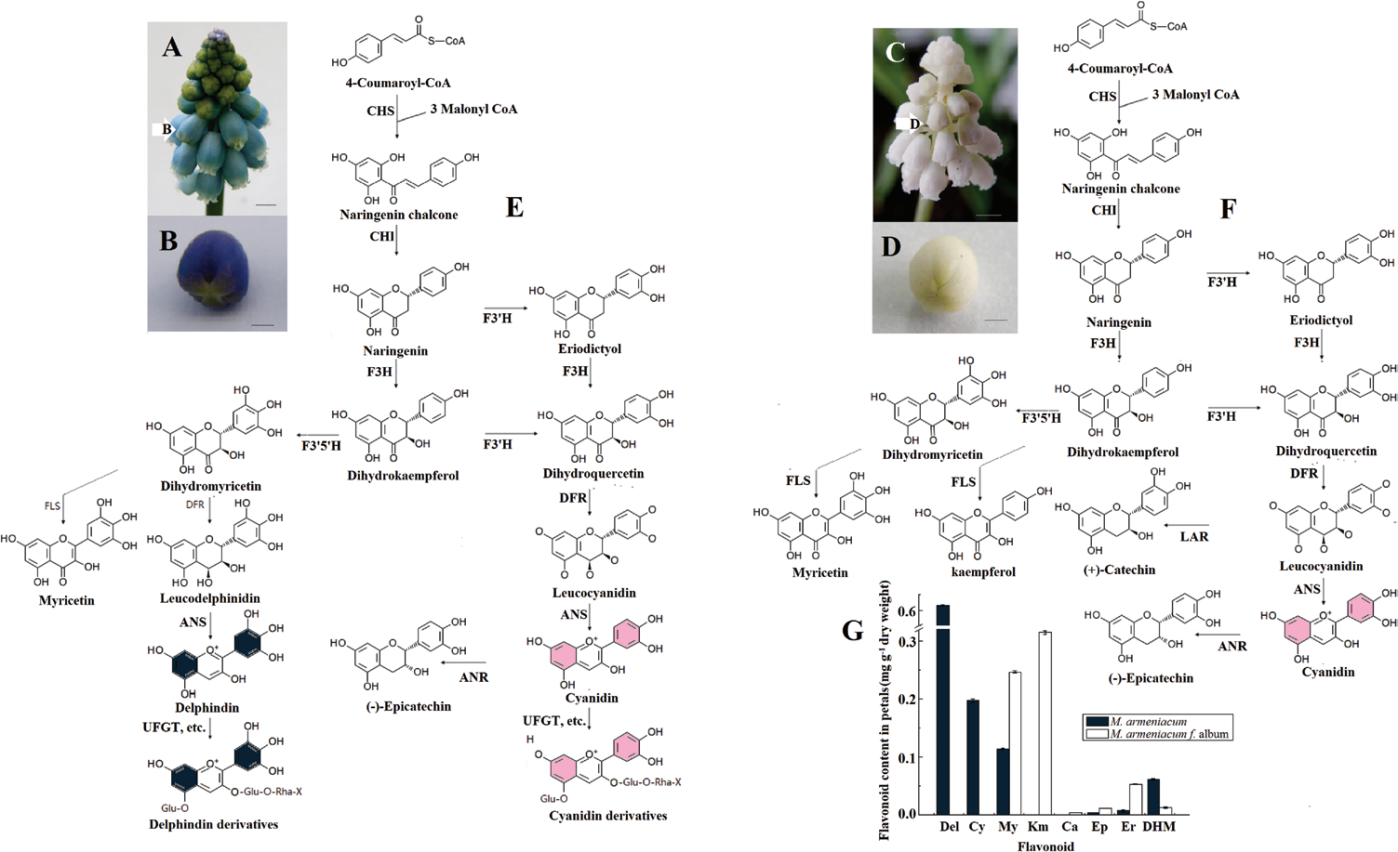
A diagram of the putative anthocyanin metabolic process in blue or white *M. armeniacum* flowers. (A) Mature inflorescence of *M. armeniacum.* Arrows represent small flower buds just before bloom. (B) Flower bud of *M. armeniacum* just before bloom used in deep sequencing. (C) Mature inflorescence of *M. armeniacum* f. album. (D) Flower bud of *M. armeniacum* f. album just before bloom used in deep sequencing. The scale bar=2mm in A and C, and 1mm in B and D. (E) The putative anthocyanin metabolic process in blue *M. armeniacum* flowers. (F) The putative anthocyanin metabolic process in white *M. armeniacum* flowers. (G) Flavonoid composition obtained by HPLC from blue and white flowers of *M. armeniacum*. ANR, anthocyanidin reductase; ANS, anthocyanidin synthase; Ca, catechin; CHI, chalcone isomerase; CHS, chalcone synthase; Cy, cyanidin; Del, delphinidin; DFR, dihydroflavonol 4-reductase; DHM, dihydromyricetin; Ep, epicatechin; Er, eriodictyol; F3H, flavanone 3-hydroxylase; F3′H, flavonoid 3′-hydroxylase; F3′5′H, flavonoid 3′5′-hydroxylase; FLS, flavonol synthase; Km, kaempferol; LAR, leucoanthocyanidin reductase; UFGT, anthocyanidin 3-*O*-glucosyltransferase. (This figure is available in colour at *JXB* online.)

The little florets just before blooming of *M. armeniacum* and its white form, *M. armeniacum* f. album were collected at 08:00h on 10 April 2012 at Xi’an Botanical Garden, Shaanxi, PR China (Fig. 1A–D). All samples were immediately frozen in liquid nitrogen and stored at –80 °C for RNA extraction and flavonoid analysis.

### Measurement of flower flavonoids

The anthocyanins were determined using high-performance liquid chromatography (HPLC) as previously described (Qi *et al.*, 2013). For extraction of other flavonoids, freeze-dried flowers were ﬁnely ground and 50mg was extracted in 500 μl of MeOH for 48h at 4 °C in darkness. After samples were centrifuged, the supernatants were transferred to fresh tubes and the pellet was resuspended and incubated in 500 μl of 1% MeOH at 4 °C for 24h, and then the supernatant was combined for further HPLC analysis. HPLC was performed as previously described (Qi *et al.*, 2013). Cyanidin, cyanidin-galactoside, dihydroquercetin, dihydrokaempferol, (+)-catechin, (–)-epicatechin, luteolin, naringenin, and quercetin were obtained from Sigma-Aldrich China (Shanghai). Standards of afzelechin, (–)-epiafzelechin, (+)-gallocatechin, and (–)-epigallocatechin were purchased from BioBioPha (Yunnan, China). The delphinidin chloride (ChromaDex, Santa Ana, CA, USA), petunidin chloride (ChromaDex), and other flavonoids such as dihydromyricetin (YiFang S&T, Tianjin, China) equivalents were used as standards for quantification. Mean values and SDs were obtained from three biological replicates.

### RNA extraction, library construction, and RNA-Seq

Total RNA of each sample was isolated using a Quick RNA isolation kit (Bioteke Corporation, Beijing, China) and then characterized on a 1% agarose gel and examined with a NanoDrop ND1000 spectrophotometer (NanoDrop Technologies, Wilmington, DE, USA). The RIN (RNA integrity number) values (>8.0) of these samples were assessed using an Agilent 2100 Bioanalyzer (Santa Clara, CA, USA). The construction of the libraries and the RNA-Seq were performed by the Biomarker Biotechnology Corporation (Beijing, China). mRNA was enriched and purified with oligo(dT)-rich magnetic beads and then broken into short fragments. Taking these cleaved mRNA fragments as templates, first- and second-strand cDNA were synthesized. The resulting cDNAs were then subjected to end-repair and phosphorylation using T4 DNA polymerase and Klenow DNA polymerase. After that, an ‘A’ base was inserted as an overhang at the 3′ ends of the repaired cDNA fragments and Illumina paired-end solexa adaptors were subsequently ligated to these cDNA fragments to distinguish the different sequencing samples. To select a size range of templates for downstream enrichment, the products of the ligation reaction were purified and selected on a 2% agarose gel. Next, PCR amplification was performed to enrich the purified cDNA template. Finally, the four libraries were sequenced using an Illumina HiSeq™ 2000.

### *De novo* transcriptome assembly and annotation

After removing those reads with only adaptor and unknown nucleotides >5%, or those that were of low quality, the clean reads were filtered from the raw reads. The clean reads were then assembled *de novo* using the Trinity platform (http://trinityrnaseq.sourceforge.net/) with the parameters of ‘K-mer=25, group pairs distance=300’ (Grabherr *et al.*, 2011). For each library, short reads were first assembled into longer contigs based on their overlap regions. Then different contigs from another transcript and their distance were further recognized by mapping clean reads back to the corresponding contigs based on their paired-end information, and thus the sequence of the transcripts was produced. Finally, the potential transcript sequences were clustered using the TGI Clustering tool to obtain uni-transcripts (Pertea *et al.*, 2003). Uni-transcripts were aligned to a series of protein databases using BLASTx (E-value ≤10^–5^), including the NCBI non-redundant (Nr), the Swiss-Prot, the Trembl, the Kyoto Encyclopedia of Genes and Genomes (KEGG) (http://www.genome.jp/kegg/kegg2.html), and gene ontology (http://wego.genomics.org.cn/cgi-bin/wego/index.pl) databases. To determine the gene coverage, the reference sequences for all three colour-related pathways were downloaded from the public databases (Supplementary Fig. S1, Supplementary Table S1 available at *JXB* online). All isoforms of all colour-related genes present in the databases examined were aligned against corresponding reference sequences using BLASTx. The deduced amino acid sequences of uni-transcripts were required to be longer than 70% of the corresponding sequences. If a uni-transcript met the criteria, it was assumed to contain a near full-length contig. If not, targeted assembly was performed to obtain even greater coverage of the respective genes. All reads in the databases examined were mapped to the reference sequences and the mapped reads were then assembled using clustering and CAP3 assembly (http://compbio.dfci.harvard.edu/tgi/software/).

### Expression annotation

To evaluate the depth of coverage, all usable reads were realigned to each uni-transcript using SOAPaligner (http://soap.genomics.org.cn/soapaligner.html), then normalized into RPKM values (reads per kb per million reads; Mortazavi *et al.*, 2008). After that, uni-transcript abundance differences between the samples were calculated based on the ratio of the RPKM values, and the false discovery rate (FDR) control method was used to identify the threshold of the *P*-value in multiple tests in order to compute the significance of the differences in transcript abundance (Benjamini and Yekutieli, 2003). Here, only uni-transcripts with an absolute value of log2 ratio ≥2 and an FDR significance score <0.001 were used for subsequent analysis.

### Gene validation and expression analysis

All the colour-related uni-transcripts were subjected to real-time quantitative PCR (q-PCR) with specific primers identified by Primer Premier software (Supplementary Table S1 at *JXB* online). cDNA synthesis and q-PCR were performed as described previously (Qi *et al.*, 2013). SYBR Green was used for detection of PCR products on a MyiQ Single-Color Real-Time Detection System (Bio-Rad). The actin gene was used as the internal control for normalization of gene expression. At least two independent biological replicates and three technical replicates of each biological replicate for each sample were analysed by q-PCR to ensure reproducibility and reliability. The correlation between expression profiles of colour-related genes measured by q-PCR and RNA-Seq was determined using the R package.

## Results and Discussion

### Major classes of colour compounds in *M. armeniacum* flowers

To examine the biochemical basis of the lack of colour phenotype of grape hyacinth, the metabolomic profiles of petals was compared, with a focus on the compounds related to colour pigmentation. As expected, blue *M. armeniacum* flowers contain two anthocyanin compounds responsible for colour pigmentation: Del and Cy. In contrast, no colour anthocyanins and no derivatives were detected in the white flowers of *M. armeniacum* f. album (Fig. 1G). Furthermore, to determine why some steps in the ABP (anthocyanin biosynthetic pathway) are blocked in white flowers, the intermediate products involved in the metabolic process and its main branches were compared. Figure 1 shows a diagram of the anthocyanin metabolic process with its core metabolites and enzymes in blue or white *M. armeniacum* flowers. Although anthocyanins were absent, petal extracts of white flowers contained all the other core metabolites involved in the process that had been detected in blue extracts (Fig. 1G). The presence of myricetin and catechin in white petals indicated that the ABP must be blocked fairly far downstream, in one of the late-acting genes such as dihydroflavonol 4-reductase (*DFR*), anthocyanidin synthase (*ANS*), or anthocyanidin 3-*O*-glucosyltransferase (*UFGT*). It is worth noting that epicatechin was detected in white flowers at a concentration three times higher than that in blue flowers (Fig. 1G). This suggests that the colour pigment Cy might be present in the white flowers, but at a level so low as to be barely detectable. Another possible explanation is that Cy exists in white grape hyacinth but only for a very short time. As soon as it formed, the unstable Cy would be converted to colourless epicatechin, which would permanently prevent Cy from changing to stable colour pigments by later glycosylation and other reactions. The compositions of common co-pigment flavonoids, such as flavones, flavonols, flavanones, caffeoyl quinic acid, and coumalic acid, were also examined to obtain a general overview of colour metabolism (Supplementary Table S2 at *JXB* online). White flowers have more typres and higher levels of the flavonoid compounds than do blue flowers, with some exceptions. Not surprisingly, the upstream flux must flow into other branches of the flavonoid metabolic route when the ABP is restrained in white flowers.

### RNA-Seq and assembly

To understand the molecular basis of flower colour polymorphism in grape hyacinth, blue flowers of *M. armeniacum* and white flowers of *M. armeniacum* f. album were used to build two libraries for high-throughput sequencing (Fig. 1B, D). The two libraries (Ma1 and Ma2) produced 2031 Mbyte and 2772 Mbyte of raw data (NCBI accessions: SRR998575 and SRR998853), respectively, from paired-end reads with a single read length of ~101bp and Q20 percentages (percentage of sequences with sequencing error rates <1%) and GC percentages of 99.45% and 50.03%, 99% and 53.12%, respectively. These data showed that the throughput and sequencing quality were high enough to warrant further analysis.

Short reads from the two libraries were assembled into 1 634 539 and 1 416 136 contigs with mean lengths of 81bp and 82bp, respectively. These were assembled into scaffolds and uni-transcripts, taking the distance of paired-end reads into account (Supplementary Table S3 at *JXB* online). All sequences were assembled to give 89 926 non-redundant uni-transcripts with a mean length of 633bp.

### Genes related to blue colour development

Genes involved in three secondary metabolic pathways (flavonoid biosynthesis, anthocyanin biosynthesis, and flavone and flavonol biosynthesis pathways) that are related to flower pigmentation were analysed using *M. armeniacum* uni-transcripts. They were searched based on standard gene names and synonyms in the combined functional annotations (Table 1). By mapping to the KEGG reference pathways, a total of 143 uni-transcripts were assigned to the three pathways (Supplementary Table S1 at *JXB* online). The data set includes annotated sequences for >88% of genes in the flavonoid biosynthesis pathway (Supplementary Fig. S1). However, only a small percentage of genes in the other two pathways was found (Supplementary Fig. S1). Possible reasons for this might be the metabolite diversification in different species. In support of this, no sequences for methoxylation genes involved in anthocyanin modification were assembled, which was consistent with the absence of methylated anthocyanin in the flowers of *M. armeniacum* (such as petunidin and malvidin; Supplementary Table S2). Therefore, it is reasonable to conclude that the ABP in grape hyacinth is unlike the pathways used in many other blue flowers in that it relies mainly on glycosylation and hydroxylation rather than methoxylation to maintain the stability of its blue pigments (Yoshida *et al.*, 2009). Moreover, an average of 71% of the full-length sequences for each of the ABP genes were obtained (Supplementary Table S1). These genes were thus the focus of further study.

**Table 1.** Candidate genes related to flower pigmentation of *M. armeniacum*

Function	Gene	Enzyme	KO id (EC no.)	No. All^*a*^	No. Up^*b*^	No. Down^*c*^
Anthocyanin biosynthesis	*CHS*	Chalcone synthase	K00660 (2.3.1.74)	17	3	4
	*CHI*	Chalcone isomerase	K01859 (5.5.1.6)	3	0	1
	*F3H*	Flavanone 3-hydroxylase	K00475 (1.14.11.9)	3	0	0
	*F3*′*H*	Flavonoid 3′-hydroxylase	K05280 (1.14.13.21)	7	0	1
	*F3*′*5*′*H*	Flavonoid 3′,5′-hydroxylase	K13083 (1.14.13.88)	4	1	1
	*DFR*	Dihydroflavonol 4-reductase	K13082 (1.1.1.219)	18	3	1
	*ANS*	Anthocyanidin synthase	K05277 (1.14.11.19)	4	0	1
	*UFGT*	Anthocyanidin 3-*O*-glucosyltransfersae	K12930 (2.4.1.115)	25	0	9
Anthocyanin modification	*UGT75C1*	Anthocyanin 5-*O*-glucosyltransferase	K12338 (2.4.1. 298)	1	0	0
	*5AT*	Anthocyanin 5-aromatic acyltransferase	K12936 (2.3.1.153)	3	0	1
	*GT1*	Anthocyanidin 5, 3-*O*-glucosyltransferase	K12938 (2.4.1.–)	12	1	0
	*3*′*GT*	UDP-glucose:anthocyanin 3′-*O*-beta-glucosyltransferase	K12939 (2.4.1.238)	4	0	0
	*5MaT1*	Anthocyanin 5-*O*-glucoside-6′′′-*O*- malonyltransferase	K12934 (2.3.1.172)	2	0	0
Flavone and flavonol biosynthesis	*FNS*	Flavone synthase	K13077 (1.14.11.22)	5	2	0
	*FLS*	Flavonol synthase	K05278 (1.14.11.23)	10	0	2
	*C12RT1*	Flavanone 7-*O*-glucoside 2′′-*O*-beta-l-rhamnosyltransferase	K13080 (2.4.1.236)	5	0	0
	*FOMT*	Flavonol 3-*O*-methyltransferase	K05279 (2.1.1.76)	6	0	0
	*CROMT2*	Myricetin *O*-methyltransferase	K13272 (2.1.1.149)	3	0	1
	*LuOMT*	Luteolin *O*-methyltransferase	\^*d*^ (2.1.1.75)	1	0	0
	*F4ST*	Flavonol 4′-sulphotransferase	K13271 (2.8.2.27)	2	0	0
	*GUSB*	beta-Glucuronidase	K01195 (3.2.1.31)	2	0	0
	*UF3GT*	Flavonol 3-*O*-glucosyltransferase	K10757 (2.4.1.91)	5	0	0
Flavanone biosynthesis	*ANR*	Anthocyanidin reductase	K08695 (1.3.1.77)	1	0	0

^*a*^ No. All, the total number of uni-transcripts analysed.

^*b*^ No. Up, the number of uni-transcripts with expression significantly up-regulated in blue flowers of *M. armeniacum* compared with in white flowers.

^*c*^ No. Down, the number of uni-transcripts with expression significantly down-regulated in blue flowers of *M. armeniacum* compared with in white flowers.

^*d*^ ¥, omission of numbers for the KO id.

### Comparison of transcriptional profiles of genes involved in anthocyanin metabolism between *M. armeniacum* and *M. armeniacum* f. album

**Fig. 2.**
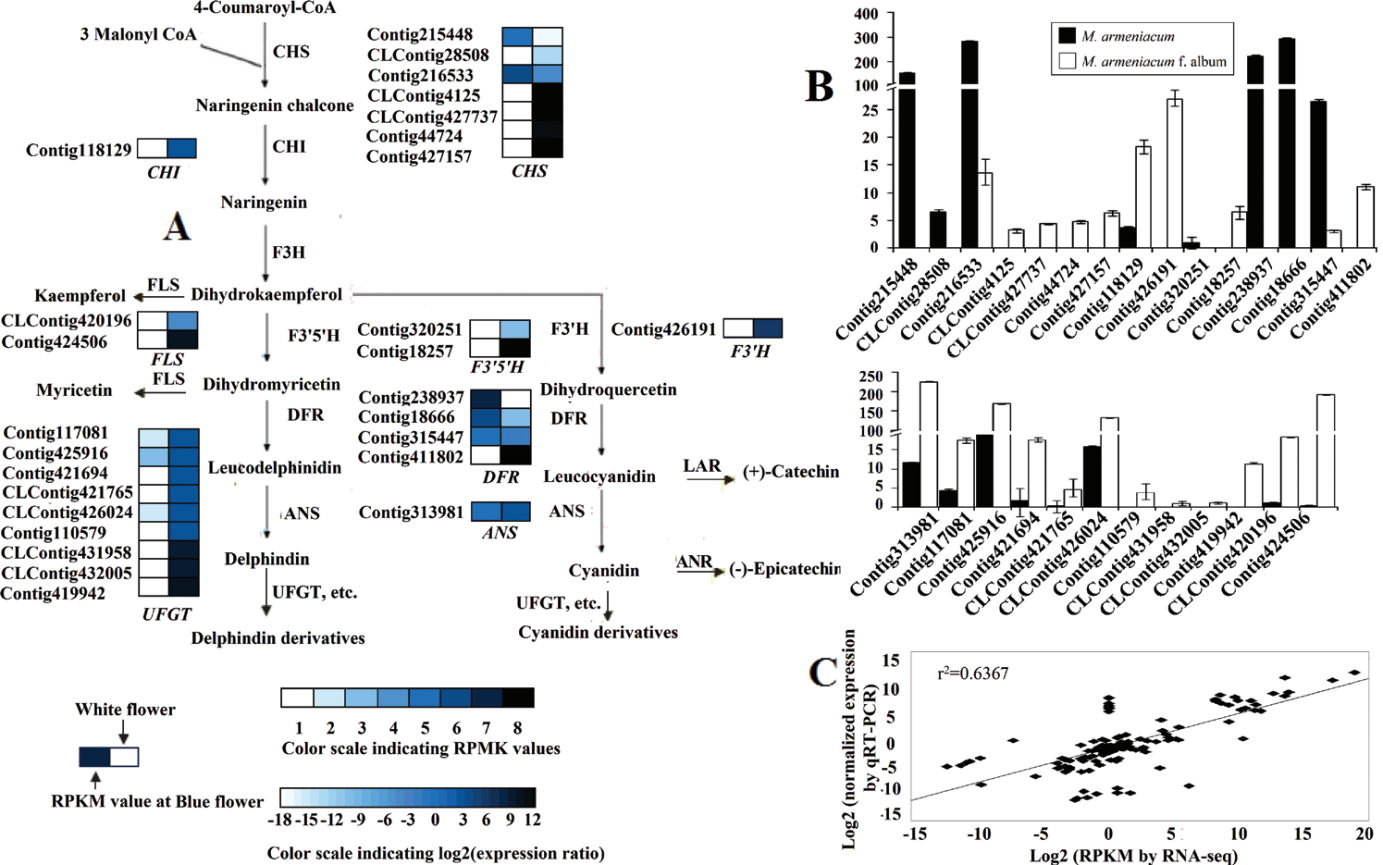
Schematic of physiological and metabolic data related to flower colour development of *M. armeniacum*. (A) A detailed part of the Del and Cy metabolic subnetwork showing the subset of nodes or metabolites that constitute the process. Enzyme names and expression patterns are indicated at the side of each step. The expression pattern of each uni-transcript is shown on two grids, with the left one representing the RPKM value of blue flowers, and the right one representing the relative log2 (expression ratio) of white flowers. The grids with eight different grey scale levels show the absolute expression magnitude of blue flowers, with the RPKM values 0–10, 10–20, 20–40, 40–80, 80–160, 160–320, 320–640, and 640–1280 represented by grey scale levels 1–8, respectively. (B) Transcript accumulation measurements of colour-related genes involved in the anthocyanin metabolic process. (C) Correlation of gene expression results obtained from q-PCR analysis and RNA-Seq for colour-related genes in blue and white flowers. ANR, anthocyanidin reductase; ANS, anthocyanidin synthase; CHI, chalcone isomerase; CHS, chalcone synthase; DFR, dihydroflavonol 4-reductase; F3H, flavanone 3-hydroxylase; F3′H, flavonoid 3′-hydroxylase; F3′5′H, flavonoid 3′5′-hydroxylase; FLS, flavonol synthase; LAR, leucoanthocyanidin reductase; UFGT, anthocyanidin 3-*O*-glucosyltransferase. (This figure is available in colour at *JXB* online.)

Previous research has demonstrated that the colour difference between white and blue flowers of *M. armeniacum* is due to the loss of flower anthocyanins (Del and Cy). The shift from blue to white requires a complete blockage of the ABP, which probably occurs in some reaction before Del and Cy are formed. Therefore, the abundance of the ABP candidate genes was compared in *M. armeniacum* and *M. armeniacum* f. album transcriptomes to find the key transcripts of blue colour metabolism. Core genes in the pathway were studied in detail, and the results demonstrated that most of the uni-transcripts with significant changes in expression level, regardless of whether they were early [chalcone isomerase (*CHI*), etc] or late genes (*ANS*, *UFGT*, etc.), showed higher transcript abundance in white flowers than in blue flowers (Fig. 2A, B). Interestingly, this result is in sharp contrast to the results of some other studies. In many cases, changes in anthocyanin accumulation have corresponded to changes in expression of genes encoding pathway enzymes (Castellarin and Gaspero, 2007; Wang *et al.*, 2010; Feng *et al.*, 2012; Yuan *et al.*, 2013). To elucidate this matter, the metabolomic profiles of blue petals were compared with those of white petals. A large quantity of flavonoid compounds was detected in white petal extracts, many of them sharing the same intermediates or enzymes with anthocyanin. For example, the contents of myricetin and kaempferol are two and three times greater, respectively, in white petals (Fig. 1G). Common enzymatic steps shared by the biosynthesis of these compounds and anthocyanins are catalysed by chalcone synthase (CHS), flavanone 3′-hydroxylase (F3′H), flavonoid 3′5′-hydroxylase (F3′5′H), etc. (Fig. 2A). This could be the reason why anthocyanin content was not correlated with the expression of anthocyanin biosynthetic genes in grape hyacinth.

### Candidates which are responsible for the loss of blue colour in grape hyacinth with white flowers

**Fig. 3.**
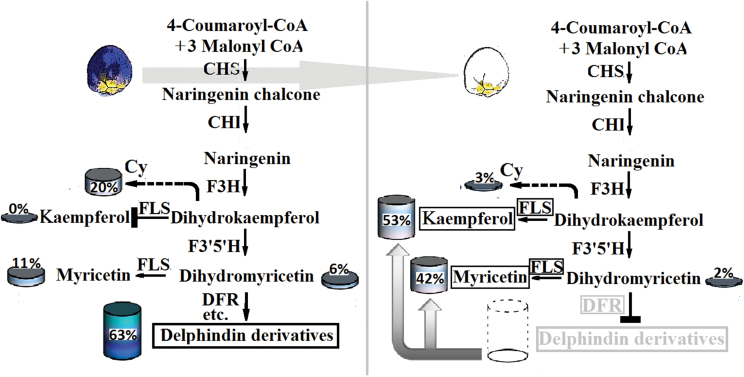
A model for the process of Del elimination in the white flowers of *M. armeniacum*. When *DFR* is suppressed, the substrates used for Del synthesis are then available for synthesis of myricetin and kaempferol. Moreover, an increase of flavonol production occurs through the up-regulation of *FLS*, furthering the process of blue pigmentation elimination in the white flowers of *M. armeniacum*. The global output from the minimal anthocyanin subnetwork in flowers of *M. armeniacum* was considered to be 100% and was used to define the relative level of each product. The black boxes indicate the genes or the compounds which had a higher relative abundance in white flowers of *M. armeniacum* than that in blue flowers. The grey boxes indicate the genes or the compounds which had a lower abundance in white flowers than that in blue flowers. CHI, chalcone isomerase; CHS, chalcone synthase; Cy, the global output from the minimal cyanidin subnetwork; DFR, dihydroflavonol 4-reductase; F3H, flavanone 3-hydroxylase; F3′5′H, flavonoid 3′5′-hydroxylase; FLS, flavonol synthase. (This figure is available in colour at *JXB* online.)

Even though most Del- and Cy-related reactions may share the same enzymes, not enough is known about how and when they catalyse the corresponding reactions. Accordingly, each event was treated independently. Of all uni-transcripts involved in the Del biosynthesis process, only three *CHS*, three *DFR*, and one *F3*′*5*′*H* homologous sequences showed significantly up-regulated expression in blue flowers; these are thought to be the flux-limiting genes leading to Del elimination in white grape hyacinth. It is generally known that CHS catalyses the first reaction for anthocyanin biosynthesis and helps to form the intermediate chalcone, the primary precursor for all classes of flavonoids (Koes *et al.*, 1989). So if CHS reactions are strongly constrained, not only anthocyanin production but also that of nearly all other flavonoids is effectively eliminated (Clark *et al.*, 2011). On the other hand, F3′5′H plays critical roles in the flavonoid biosynthetic pathway, and catalyses the hydroxylation of the B-ring of flavonoids and is necessary to biosynthesize Del (violet to blue)-based anthocyanins (Tanaka and Brugliera, 2013). It was expected that, in the event that the minimal Del path was cut off from F3′5′H, myricetin-related flavonols would be removed along with Del. In fact, however, a great deal of myricetin was found in white flowers, more than twice as much as in blue flowers (Fig. 1G). Yet this is not a satisfactory explanation for the lack of Del in white grape hyacinth. Hence *DFR*, a crucial later gene for anthocyanin formation, was considered. As shown in Fig. 1, DFR reduces dihydroflavonols to colouress leucoanthocyanidins, which are catalysed by ANS to coloured anthocyanidins. No products of the Del synthesis route that occur after dihydromyricetin (the substrate for the DFR enzyme) were detected in white flowers (Fig. 3), suggesting that *DFR* was the most likely target for Del suppression in *M. armeniacum* f. album. It is noteworthy that the transcripts of three *DFR*-like sequences showed significantly higher levels of gene transcripts in blue flowers than in white flowers, in some cases >1000 times higher (Fig. 2A). Although this was unexpected, it is a reasonable explanation for the fact that the Del synthesis reactions are constrained to zero. Additionally, the dihydroflavonols represent a branch point in flavonoid biosynthesis, being the intermediates in the production of both the coloured anthocyanins, through DFR, and the colourless flavonols, through flavonol synthase (FLS) (Davies *et al.*, 2003). As a result of the competition for substrate (dihydroflavonols), the up-regulation of FLS and flavonols might be closely accompanied by a decrease in DFR and anthocyanin accumulation. In support of this, inhibition of FLS production through the introduction of an FLS antisense RNA construct led to anthocyanin production and gave the white-flowered petunia a novel pink hue (Davies *et al.*, 2003). In the present study, the abundance of myricetin (a downstream flavonol product of dihydromyricetin) and that of two FLS-like sequences were far greater in white grape hyacinth than in the blue-flowered strain, confirming the hypothesis by another approach. Combining the information with data from HPLC, it could be inferred that *DFR* might be the target gene for the loss of blue pigmentation (Del) in white grape hyacinth. In addition, strong competition between FLS and DFR for common dihydromyricetin substrates might partially block the synthesis of Del and cause the production of other flavonoid compounds such as myricetin, thereby furthering the process of elimination of blue pigmentation and shifting the flavonol:anthocyanin ratio in *M. armeniacum*.

### Reasons for loss of red Cy accumulation in white-flowered grape hyacinth

**Fig. 4.**
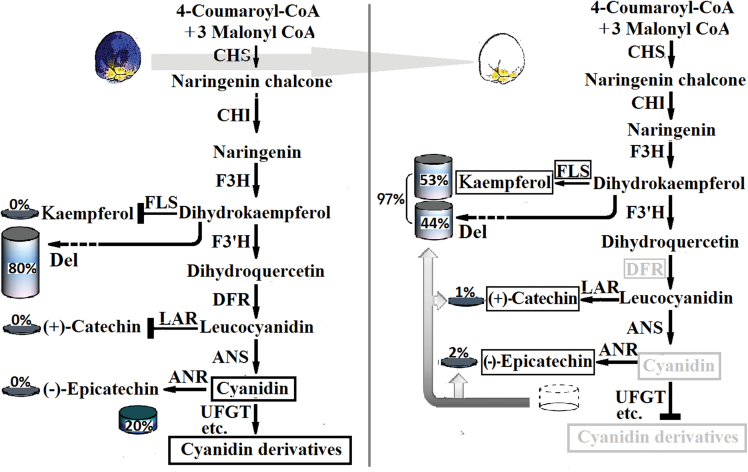
A model for Cy elimination in white flowers of *M. armeniacum*. The fluxes through Cy metabolism were limited. The multishunt process in downstream reactions further promoted Cy turnover and degradation in white flowered grape hyacinth. The global output from the minimal anthocyanin subnetwork in flowers of *M. armeniacum* was considered as 100% and was used to define the relative level of each product. The black boxes indicate the genes or the compounds which had a higher relative abundance in white flowers of *M. armeniacum* than that in blue flowers. The grey boxes indicate the genes or the compounds which had a lower abundance in white flowers than that in blue flowers. ANR, anthocyanidin reductase; ANS, anthocyanidin synthase; CHI, chalcone isomerase; CHS, chalcone synthase; Del, the global output from the minimal delphinidin subnetwork; DFR, dihydroflavonol 4-reductase; F3H, flavanone 3-hydroxylase; F3′H, flavonoid 3′-hydroxylase; FLS, flavonol synthase; LAR, leucoanthocyanidin reductase; UFGT, anthocyanidin 3-*O*-glucosyltransferase. (This figure is available in colour at *JXB* online.)

To select the target genes for Cy suppression in grape hyacinth, the expression and metabolomic profiles of blue and white petals were compared in whole Cy metabolic reactions. The presence of catechin and epicatechin in white petals indicated that the red Cy must be present in the white flowers, even if only for a very short time or in a very small quantity, hinting at a complex metabolic mechanism underlying the loss of Cy pigmentation. There may be multiple reasons for this phenomenon. First, *DFR* and *FLS* were good candidates for the limitation of Cy accumulation, as discussed earlier. When *FLS* is up-regulated, the substrates used for Cy synthesis are then available for synthesis of kaempferol in white flowers (Fig. 4). The down-regulation of *DFR* could decreased Cy production, but obviously it cannot produce a complete blockage of the process on its own. Secondly, the metabolism of Del plays a particularly important role in the flower coloration system of *M. armeniacum*, whereas the metabolism of Cy is less significant (Fig. 4). In blue flowers, the total content of Del (blue) was three times higher than that of Cy (red), which might also explain why blue is the predominant colour hue in *M. armeniacum* flowers. Even in white flowers, the 44% yield from the Del metabolic pathway was much higher than the 3% yield from the Cy metabolic pathway (Fig. 4). The low level of productive forces might limit the flux through Cy metabolism in grape hyacinth and explain the small amounts of Cy that accumulate in the white flowers. Thirdly, as is known, the last product before Cy formation is leucyanidin, which can generate two different products, colourless catechin and red Cy, in reactions catalysed by leucoanthocyanidin reductase (LAR) and ANS, respectively. In *M. armeniacum*, catechin was detected only in white flowers and not in blue flowers (Fig. 4). Therefore, it could be concluded that the alteration in competition from LAR for the substrate might redirect Cy biosynthesis towards catechin and further restrict the flux through its subsequent biosynthesis process. Fourthly, the next step after Cy formation should convert unstable anthocyanin to stable coloured compounds, but the white flowers contain increased concentrations of epicatechin and undetectable levels of Cy (Fig. 4). It is suggested that the low amounts of Cy might be reduced to colourless epicatechin by anthocyanidin reductase (ANR) and thus redirect anthocyanin biosynthesis away from the production of stable Cy-based pigments. Above all, the limitation of flux in upstream reactions and the multishunt process in downstream reactions led to the process of elimination of red pigmentation in the white flowers of *M. armeniacum*.

Recently, Clark *et al.* (2011) considered the advantages of targeting *DFR* in order to eliminate floral pigmentation: the production of only a few compounds is affected; it does not operate too late in the ABP pathway; it is more essential for anthocyanin production than are other earlier genes, etc. It seems to be a very attractive means, for both plants and breeders, by which to change flower colour from blue to white by the down-regulation of a single *DFR*. Nevertheless, it seems that such loss-of-colour adaptations are relatively unconstrained in different species because they can be achieved in many ways. For example, the mutation of a single *CHS* enzyme is often observed. It leads to white flower lines in the petunia (Saito *et al.*, 2006; Spitzer *et al.*, 2007), violet (Hemleben *et al.*, 2004), and arctic mustard flower (Dick *et al.*, 2011). Blocking an early-acting gene such as *CHS* could be more efficient. Perhaps this is why *CHS* mutation is the most common means of producing loss of colour in the literature (Clark and Verwoerd, 2011). Another common reason for pigmentation loss is the absence of more than one enzyme in the ABP, such as ANS and DFR (Ma *et al.*, 2004; Bogs *et al.*, 2007; Clark and Verwoerd, 2011). Recent research has described many new ways to determine the lack of colour phenotype by regulating the branching point of anthocyanin biosynthesis. For instance, inhibition of ANR and consequent LAR production by the transient suppression of the *FcMYB1* gene in white strawberry fruit leads to increased concentrations of anthocyanins and undetectable levels of flavan-3-ols (Salvatierra *et al.*, 2013). Similarly, introduction of apple *ANR* genes into tobacco inhibits expression of both *CHI* and *DFR* genes in flowers, finally leading to loss of anthocyanin (Han *et al.*, 2012). Here, a new hypothesis is proposed explaining a lack of colour phenotype of grape hyacinth flowers. The truth of the matter is probably more complex than what has been described here, the elucidation of which could be an interesting and challenging subject.

## Supplementary data

Supplementary data are available at *JXB* online.

Figure S1. KEGG reference mappings for flavonoid synthesis, anthocyanin biosynthesis, and flavone and flavonol biosynthesis pathways.

Table S1. List of relative uni-transcripts in the three secondary metabolic pathways in the *M. armeniacum* transcriptome.

Table S2. The contents of flavonoids in flower petals of *M. armeniacum*.

Table S3. Length and gap distribution of contigs, scaffolds, and uni-transcripts from each library of *M. armeniacum*.

Supplementary Data

## Abbreviations:

ABP: anthocyanin biosynthetic pathway
ANR: anthocyanidin reductase
ANS: anthocyanidin synthase
BLAST: Basic local alignment search tool
CHS: chalcone synthase
Cy: cyanidin
Del: delphinidin
DFR: dihydroflavonol 4-reductase
F3′H: flavanone 3′-hydroxylase
F3′5′H: flavonoid 3′5′-hydroxylase
FDR: false discovery rate
FLS: flavonol synthase
HPLC: high-performance liquid chromatography
KEGG: the Kyoto Encyclopedia of Genes and Genomes
LAR: leucoanthocyanidin reductase
NCBI: National Center for Biotechnology Information
Nr: NCBI non-redundant database
q-PCR: real-time quantitative PCR
RNA-Seq: RNA sequencing
RPKM: reads per kb per million reads
SOAP: short oligonucleotide analysis package
UFGT: anthocyanidin 3-*O*-glucosyltransferase.

## References

[CIT0001] Benjamini Y Yekutieli D 2001 The control of the false discovery rate in multiple testing under dependency. Annals of Statistics 29, 1165–1188

[CIT0002] Bogs J Jaffe FW Takos AM Walker AR Robinson SP 2007 The grapevine transcription factor VvMYBPA1 regulates proanthocyanidin synthesis during fruit development. Plant Physiology 143, 1347–1361 17208963 10.1104/pp.106.093203 PMC1820911

[CIT0003] Castellarin SD Gaspero GD 2007 Transcriptional control of anthocyanin biosynthetic genes in extreme phenotypes for berry pigmentation of naturally occurring grapevines. BMC Plant Biology 7, 46 17760970 10.1186/1471-2229-7-46 PMC2147006

[CIT0004] Clark ST Verwoerd WS 2011 A systems approach to identifying correlated gene targets for the loss of colour pigmentation in plants. BMC Bioinformatics 12, 343 21849042 10.1186/1471-2105-12-343 PMC3180701

[CIT0005] Davies KM Schwinn KE Deroles SC Manson DG Lewis DH Bloor SJ Bradley JM 2003 Enhancing anthocyanin production by altering competition for substrate between flavonol synthase and dihydroflavonol 4-reductase. Euphytica 131, 259–268

[CIT0006] Dick CA Buenrostro J Butler T Carlson ML Kliebenstein DJ Whittall JB 2011 Arctic mustard flower color polymorphism controlled by petal-specific downregulation at the threshold of the anthocyanin biosynthetic pathway. PLoS One 6, e18230 21490971 10.1371/journal.pone.0018230 PMC3072389

[CIT0007] Feng C Chen M Xu CJ Bai L Yin XR Li X Allan AC Ferguson IB Chen KS 2012 Transcriptomic analysis of Chinese bayberry (*Myrica rubra*) fruit development and ripening using RNA-Seq. BMC Genomics 13, 19 22244270 10.1186/1471-2164-13-19 PMC3398333

[CIT0008] Grabherr MG Haas BJ Yassour M 2011 Full-length transcriptome assembly from RNA-Seq data without a reference genome. Nature Biotechnology 29, 644–652 10.1038/nbt.1883 PMC3571712 21572440

[CIT0009] Grotewold E 2006 The genetics and biochemistry of floral pigments. Annual Review of Plant Biology 57, 761–780 10.1146/annurev.arplant.57.032905.105248 16669781

[CIT0010] Han YP Vimolmangkang S Soria-Guerra RE Korban SS 2012 Introduction of apple *ANR* genes into tobacco inhibits expression of both *CHI* and *DFR* genes in flowers, leading to loss of anthocyanin. Journal of Experimental Botany 63, 2437–2447 22238451 10.1093/jxb/err415 PMC3346214

[CIT0011] Hemleben V Dressel A Epping B Lukačin R Martens S Austin M 2004 Characterization and structural features of a chalcone synthase mutation in a white-flowering line of *Matthiola incana* R. Br. (*Brassicaceae*). Plant Molecular Biology 55, 455–465 15604692 10.1007/s11103-004-1125-y

[CIT0012] Koes RE Spelt CE Elzen PJMV Mol JNM 1989 Cloning and molecular characterization of the chalcone synthase multigene family of *Petunia hybrida* . Gene 81, 245–257 2806915 10.1016/0378-1119(89)90185-6

[CIT0013] Ma H Zhao X Yuan Y Zeng A 2004 Decomposition of metabolic network into functional modules based on the global connectivity structure of reaction graph. Bioinformatics 20, 1870–1876 15037506 10.1093/bioinformatics/bth167

[CIT0014] Mori S Asano S Kobayashi H Nakano M 2002 Analyses of anthocyanidins and anthocyanins in flowers of *Muscari* spp. Bulletin of the Faculty of Agriculture 55, 13–18

[CIT0015] Mortazavi A Williams BA McCue K Schaeffer L Wold B 2008 Mapping and quantifying mammalian transcriptomes by RNA-Seq. Nature Methods 5, 621–628 18516045 10.1038/nmeth.1226 PMC13303166

[CIT0016] Pertea G Huang XQ Liang F 2003 TIGR gene indices clustering tools (TGICL): a software system for fast clustering of large EST datasets. Bioinformatics 19, 651–652 12651724 10.1093/bioinformatics/btg034

[CIT0017] Qi YY Lou Q Li HB Yue J Liu YL Wang YJ 2013 Anatomical and biochemical studies of bicolored flower development in *Muscari latifolium* . Protoplasma 250, 1273–1281 23677687 10.1007/s00709-013-0509-8

[CIT0018] Saito R Fukuta N Ohmiya A Itoh Y Ozeki Y Kuchitsu K Nakayama M 2006 Regulation of anthocyanin biosynthesis involved in the formation of marginal picotee petals in *Petunia* . Plant Science 170, 828–834

[CIT0019] Salvatierra A Pimente P Moya-León MA Herrera R 2013 Increased accumulation of anthocyanins in *Fragaria chiloensis* fruits by transient suppression of *FcMYB1* gene. Phytochemistry Letters 90, 25–36 10.1016/j.phytochem.2013.02.016 23522932

[CIT0020] Spitzer B Zvi MMB Ovadis M 2007 Reverse genetics of floral scent: application of tobacco rattle virus-based gene silencing in *Petunia* . Plant Physiology 145, 1241–1250 17720754 10.1104/pp.107.105916 PMC2151718

[CIT0021] Tanaka Y Brugliera F 2013 Flower colour and cytochromes P450. Philosophical Transactions of theRoyal Society B: Biological Sciences 368, 20120432 10.1098/rstb.2012.0432 PMC3538422 23297355

[CIT0022] Tanaka Y Sasaki N Ohmiya A 2008 Biosynthesis of plant pigments: anthocyanins, betalains and carotenoids. The Plant Journal 54, 733–749 18476875 10.1111/j.1365-313X.2008.03447.x

[CIT0023] Wang KL Bolitho K Grafton K Kortstee A Karunairetnam S McGhie TK Espley RV Hellens RP Allan AC 2010 An R2R3 MYB transcription factor associated with regulation of the anthocyanin biosynthetic pathway in *Rosaceae.* BMC Plant Biology 10, 50 20302676 10.1186/1471-2229-10-50 PMC2923524

[CIT0024] Yoshida K Mori M Kondo T 2009 Blue flower color development by anthocyanins: from chemical structure to cell physiology. Natural Product Reports 26, 884–915 19554240 10.1039/b800165k

[CIT0025] Yuan Y Ma XH Shi YM Tang DQ 2013 Isolation and expression analysis of six putative structural genes involved in anthocyanin biosynthesis in *Tulipa fosteriana* . Scientia Horticulturae 153, 93–102

