# Supplementary material for: Transcriptome sequencing and metabolite analysis reveals the role of delphinidin metabolism in flower colour in grape hyacinth
Source: J Exp Bot. 2014 Apr 30;65(12):3157–64. doi: 10.1093/jxb/eru168 (PMC4071837; doi:10.1093/jxb/eru168)
Supplement: Supplementary Data [file supp_65_12_3157__index.html]

Transcriptome sequencing and metabolite analysis reveals the role of delphinidin metabolism in flower colour in grape hyacinth — Transcriptome sequencing and metabolite analysis reveals the role of delphinidin metabolism in flower colour in grape hyacinth — Supplementary Data 

# Transcriptome sequencing and metabolite analysis reveals the role of delphinidin metabolism in flower colour in grape hyacinth

## Supplementary Data

Data files

**Files in this Data Supplement:**

- Supplementary Data - Supplementary Data
- Supplementary Data - Supplementary Data
- Supplementary Data - Supplementary Data
- Supplementary Data - Supplementary Data
