# Supplementary figures and images for: Transcriptome sequencing and metabolite analysis reveals the role of delphinidin metabolism in flower colour in grape hyacinth
Source: J Exp Bot. 2014 Apr 30;65(12):3157–64. doi: 10.1093/jxb/eru168 (PMC4071837; doi:10.1093/jxb/eru168)

## FLAVONOID BIOSYNTHESIS

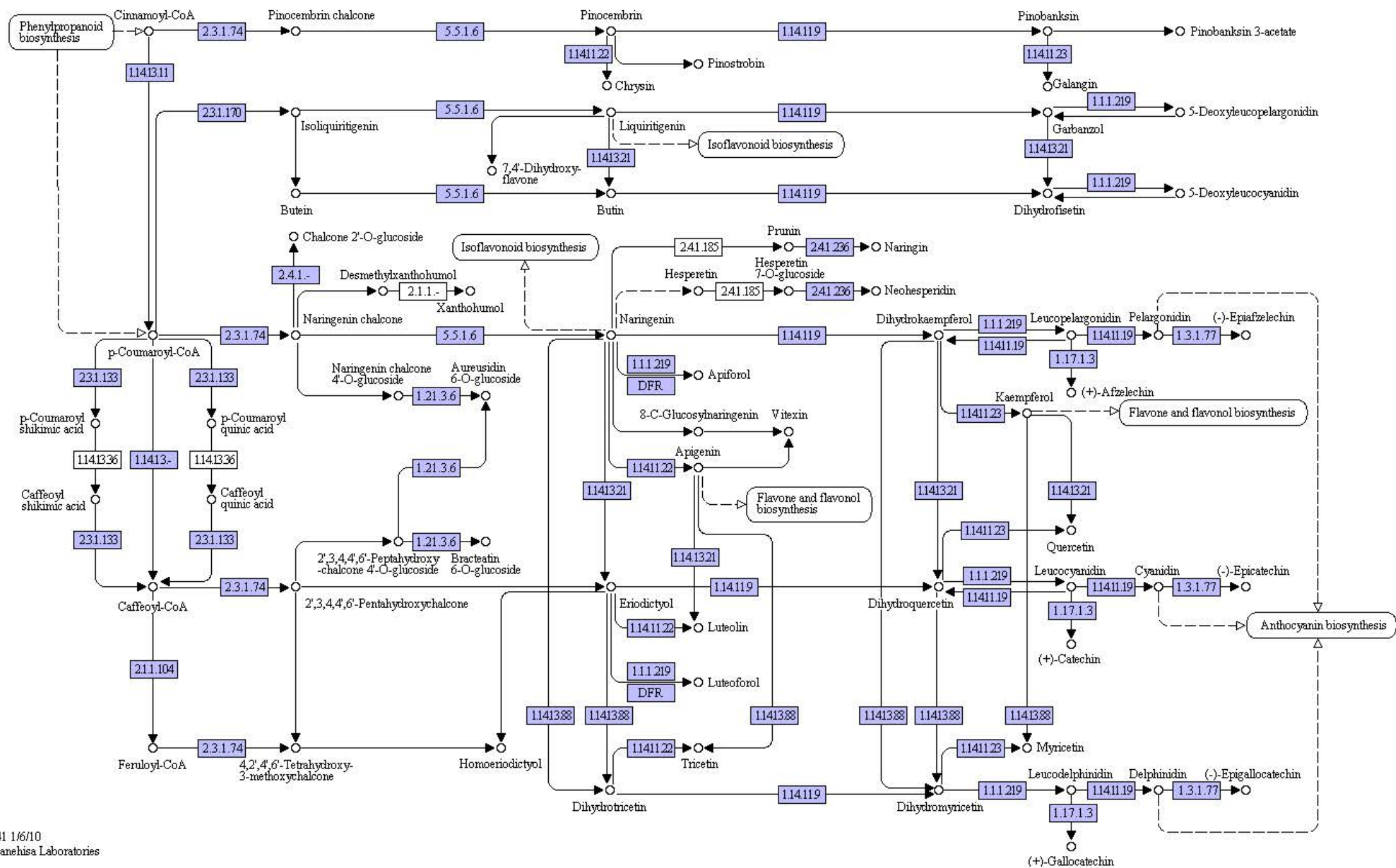

#### Flavonoid biosynthesis

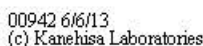

FLAVONE AND FLAVONOL BIOSYNTHESIS

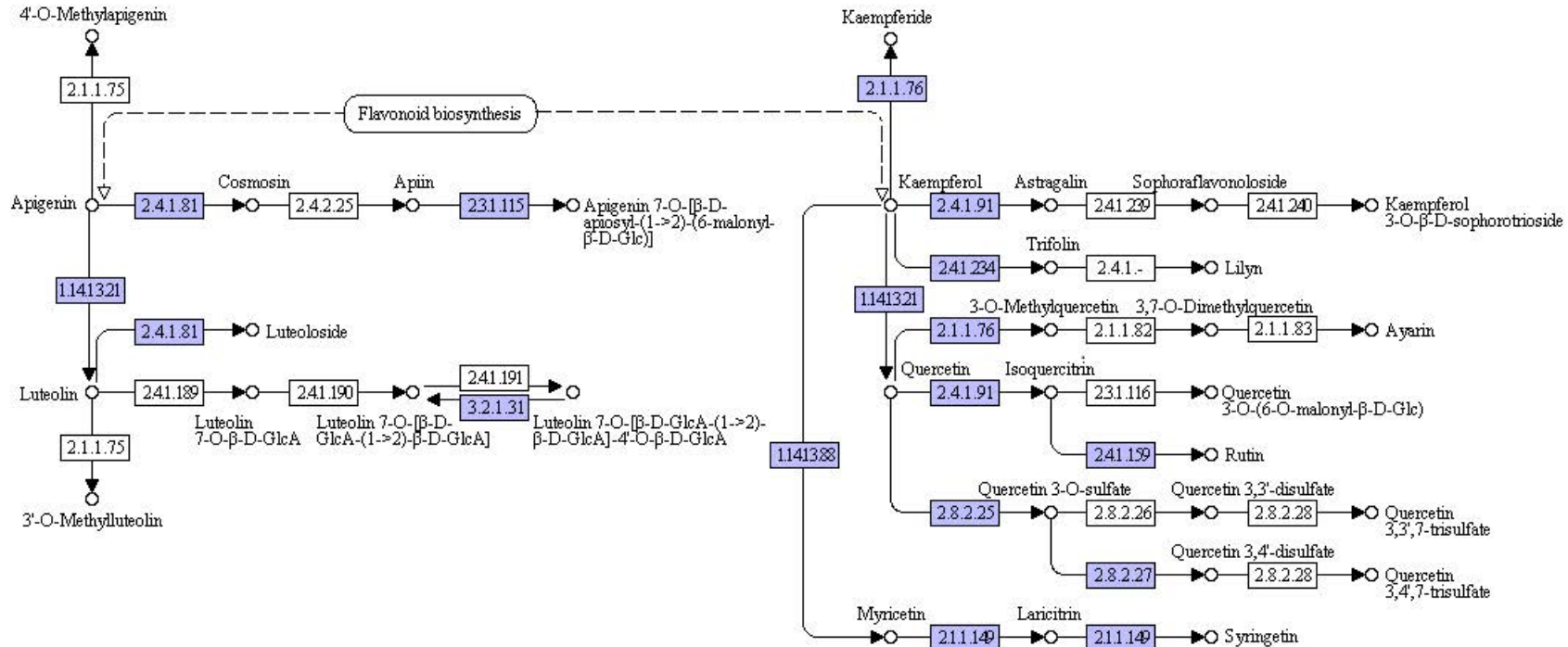

Supplement: Supplementary Data [file supp_eru168_jexbot113068_file004.pdf]
